# Implementing machine learning in bipolar diagnosis in China

**DOI:** 10.1038/s41398-019-0638-8

**Published:** 2019-11-18

**Authors:** Yantao Ma, Jun Ji, Yun Huang, Huimin Gao, Zhiying Li, Wentian Dong, Shuzhe Zhou, Yue Zhu, Weimin Dang, Tianhang Zhou, Haiqing Yu, Bin Yu, Yuefeng Long, Long Liu, Gary Sachs, Xin Yu

**Affiliations:** 1 Peking University Sixth Hospital, Beijing, China; 2 Peking University Institute of Mental Health, Beijing, China; 3 NHC Key Laboratory of Mental Health (Peking University), Beijing, China; 4 National Clinical Research Center for Mental Disorders (Peking University Sixth Hospital), Beijing, China; 5 College of Computer Science and Technology, Qingdao University, Qingdao, China; 6 Beijing Wanling Pangu Science and Technology Ltd, Beijing, China; 7 Department of Surgery, Stanford University, Stanford, CA 94305 USA; 8 Qingdao University Medical College, Qingdao, China; 9 State Key Laboratory of Industrial Control Technology, College of Control Science and Engineering, Zhejiang University, Hangzhou, China; 10 Harvard University Massachusetts General Hospital, Boston, MA USA

**Keywords:** Diagnostic markers, Bipolar disorder, Bipolar disorder, Diagnostic markers

## Abstract

Bipolar disorder (BPD) is often confused with major depression, and current diagnostic questionnaires are subjective and time intensive. The aim of this study was to develop a new Bipolar Diagnosis Checklist in Chinese (BDCC) by using machine learning to shorten the Affective Disorder Evaluation scale (ADE) based on an analysis of registered Chinese multisite cohort data. In order to evaluate the importance of each item of the ADE, a case-control study of 360 bipolar disorder (BPD) patients, 255 major depressive disorder (MDD) patients and 228 healthy (no psychiatric diagnosis) controls (HCs) was conducted, spanning 9 Chinese health facilities participating in the Comprehensive Assessment and Follow-up Descriptive Study on Bipolar Disorder (CAFÉ-BD). The BDCC was formed by selected items from the ADE according to their importance as calculated by a random forest machine learning algorithm. Five classical machine learning algorithms, namely, a random forest algorithm, support vector regression (SVR), the least absolute shrinkage and selection operator (LASSO), linear discriminant analysis (LDA) and logistic regression, were used to retrospectively analyze the aforementioned cohort data to shorten the ADE. Regarding the area under the receiver operating characteristic (ROC) curve (AUC), the BDCC had high AUCs of 0.948, 0.921, and 0.923 for the diagnosis of MDD, BPD, and HC, respectively, despite containing only 15% (17/113) of the items from the ADE. Traditional scales can be shortened using machine learning analysis. By shortening the ADE using a random forest algorithm, we generated the BDCC, which can be more easily applied in clinical practice to effectively enhance both BPD and MDD diagnosis.

## Introduction

Bipolar disorder (BPD) is characterized by recurrent depression and mania/hypomania^1^. Difficulties and delays in the diagnosis of BPD impede the effective treatment of patients. BPD is prone to misdiagnosis as major depressive disorder (MDD). Despite being one of the 10 most debilitating noncommunicable diseases^2,3^, BPD is misdiagnosed as recurrent MDD in 60% of patients seeking treatment for depression^4^. In particular, the recent 3rd national Chinese Mental Health Survey (CMHS) reported that the 12-month prevalence rates of both BPD and MDD had increased to as high as 4.5%, while the recognition rate of BPD versus current major depressive episodes (MDEs) was as high as 39.9% according to the BRIDGE-China study^5,6^.

Therefore, there is an urgent need to improve the early diagnosis of BPD, especially in terms of distinguishing patients with BPD from those with MDD. In light of the current large number of domestic patient diagnoses, easier and targeted diagnostic evaluation tools are needed.

The affective disorder evaluation (ADE)^7^ was designed in 2003 as a confidence estimation tool to guide psychiatrists in diagnosing patients and developing treatment plans. The ADE is neither a screening instrument nor a self-report measure but provides a systematic process that helps psychiatrists apply their full assessment to judge the confidence of bipolar diagnosis. Psychometric results from the use of the ADE in routine clinical practice have been published in Russia^8^, the United States^9^, and China^10^, and the output measure is known as the bipolarity index (BPx). However, the ADE contains 145 questions and usually requires 45–90 min to derive the BPx score and form a diagnostic impression. Thus, the ADE is too time consuming for use in clinical practice because psychiatrists cannot allocate that amount of time to each visit, considering the heavy load of patients seeing psychiatrists in China.

Machine learning algorithms can effectively leverage cohort data to generate classifiers and measure the sensitivity and specificity of parameters with respect to diagnostic validity and similarity to the original and revised diagnostic evaluation tools. Machine learning algorithms have already been implemented to shorten many scales, such as the Autism Diagnostic Observation Schedule-Generic (ADOS) for autism diagnosis^11^ and the Social Responsiveness Scale (SRS) for behavioral distinction between autism and attention-deficit/hyperactivity disorder (ADHD)^12^.

In this study, to develop a shortened version of the ADE feasible for use in clinical practice, which we named the Bipolar Diagnosis Checklist in Chinese (BDCC), we used a machine learning algorithm to shorten the original ADE based on a retrospective analysis of the Comprehensive Assessment and Follow-up Descriptive Study on Bipolar Disorder (CAFÉ-BD) data.

## Materials and methods

### Data sample

The included MDD (*N* = 255) and BPD (*N* = 360) subjects were outpatients or inpatients at a health facility affiliated with the CAFÉ-BD. The healthy control (HC) (*N* = 228) subjects were recruited among people who responded to flyers distributed near the participating health centers.

The CAFÉ-BD is a collaborative study of nine health centers in China with the goal of implementing a set of standardized intake procedures among six psychiatric hospitals and the mental health departments of three general hospitals. The details of the CAFÉ-BD can be found at http://ClinicalTrials.gov under the identifier NCT02015143. Ethical approval was obtained from all participating centers.

### Sample and assessment procedure

All subjects signed written informed consent and were then initially evaluated by CAFÉ-BD researchers using the Mini-International Neuropsychiatric Interview 5.0 (MINI 5.0)^13–15^. During subsequent visits, different CAFÉ-BD investigators independently completed the ADE and recorded the resulting bipolarity index (BPx). According to the MINI diagnosis results, patients were divided into different groups based on the presence or absence of each mood disorder. The details of the BPx and the MINI as well as the inclusion and exclusion criteria for MDD and BPD can be found in our previous work^10^. Each site enrolled at least 37 BPD and 25 MDD patients^10^. HC subjects matched with the MDD and BPD subjects by gender, age and education were then recruited. In this way, each site enrolled at least 45 HCs, of which 23 were matched with the BPD patients and 22 were matched with the MDD patients^10^.

### Machine learning

First, according to expert suggestions on our previous ADE study, we reduced the initial 145 items of the ADE to 113 items by deleting items that had no diagnostic relevance, such as type of medical insurance. Then, five machine learning algorithms were implemented to analyze the aforementioned data using the 113 questions in the ADE as features and MDD, BPD, and HC as prediction classes. Our machine learning pipeline was initialized by randomly splitting the entire data set into 10 stratified subsets, where each subset consisted of 10% of the MDD data (*N* = 255), BPD data (*N* = 360), and HC data (*N* = 228). Then, 10-fold cross-validation was implemented using these subsets, where each cross-validation trial iteratively utilized one subset for testing and the remaining nine subsets for training. For each trial, feature ranking was calculated using the 9-fold training set. All features were ranked based on the minimal-redundancy-maximal-relevance (mRMR) mutual information criterion^16^. Then, forward feature selection was performed using the previously obtained ranks to train and test the five machine learning algorithms, with parameter tuning for each choice of features. This process was iteratively implemented for every 10 cross-validation trials, resulting in an average area under the receiver operating characteristic (ROC) curve (AUC) for each model calculated over 100 trials. All machine learning analyses were performed in R. The support vector regression (SVR) algorithm was implemented using the kernlab package with Weston and Watkins’ native multiclass formulation^17^ and a radial basis function (RBF) kernel^18^. LASSO was implemented using the glmnet package^19^, logistic regression was implemented using the net package^20^, and linear discriminant analysis (LDA) was implemented using the MASS package^21^.

## Results

For each trial in the 10-fold cross-validation, the mutual information feature ranking was calculated over the 9-fold training set. In each of these 10 trials, the rankings were the same; the top 17 features are shown in Table 1.

**Table 1:** The top 17 features ranked by mRMR.

Rank	BDCC Items	mRMR
1	Over the past 2 weeks, how many days have you had any severe abnormal mood elevation?	0.229
2	Other features of past episodes of depression: sudden onset?	0.037
3	Dysthymia: depressed more days than not for >2 years?	0.037
4	Over the past 2 weeks, how many days have you had lowered interest in most activities or found that you could not enjoy even pleasurable activities most of the day?	0.031
5	How old were you when you were first treated for depression?	0.031
6	Rate associated symptoms for the past week: guilt	0.010
7	Rate associated symptoms for the past week: life not worth living (LNWL)	0.007
8	Rate associated symptoms for the past week: flight of ideas (FOI)/racing thoughts	0.005
9	Past psychiatric history: suicide attempt	0.005
10	Over the past year, how many days have you had any abnormal anxiety?	0.002
11	Past depression: other features of past episodes of depression: anger attacks	0.002
12	Over the past 2 weeks, how many days have you had any abnormal severe irritability?	0.000
13	Abnormal mood elevation (lifetime): during the most severe episode identified above, were there any times when your mood was euphoric?	−0.001
14	Over the past 2 weeks, how many days have you been depressed most of the day?	−0.003
15	Rate associated symptoms for the past week: sleep anhedonia	−0.004
16	Rate associated symptoms for the past week: psychomotor agitation (PMA)	−0.005
17	Past depression: other features of past episodes of depression: feelings of worthlessness	−0.005

*LNWL* life not worth living, *FOI* flight of ideas, *PMA* psychomotor agitation

**Fig. 1:**
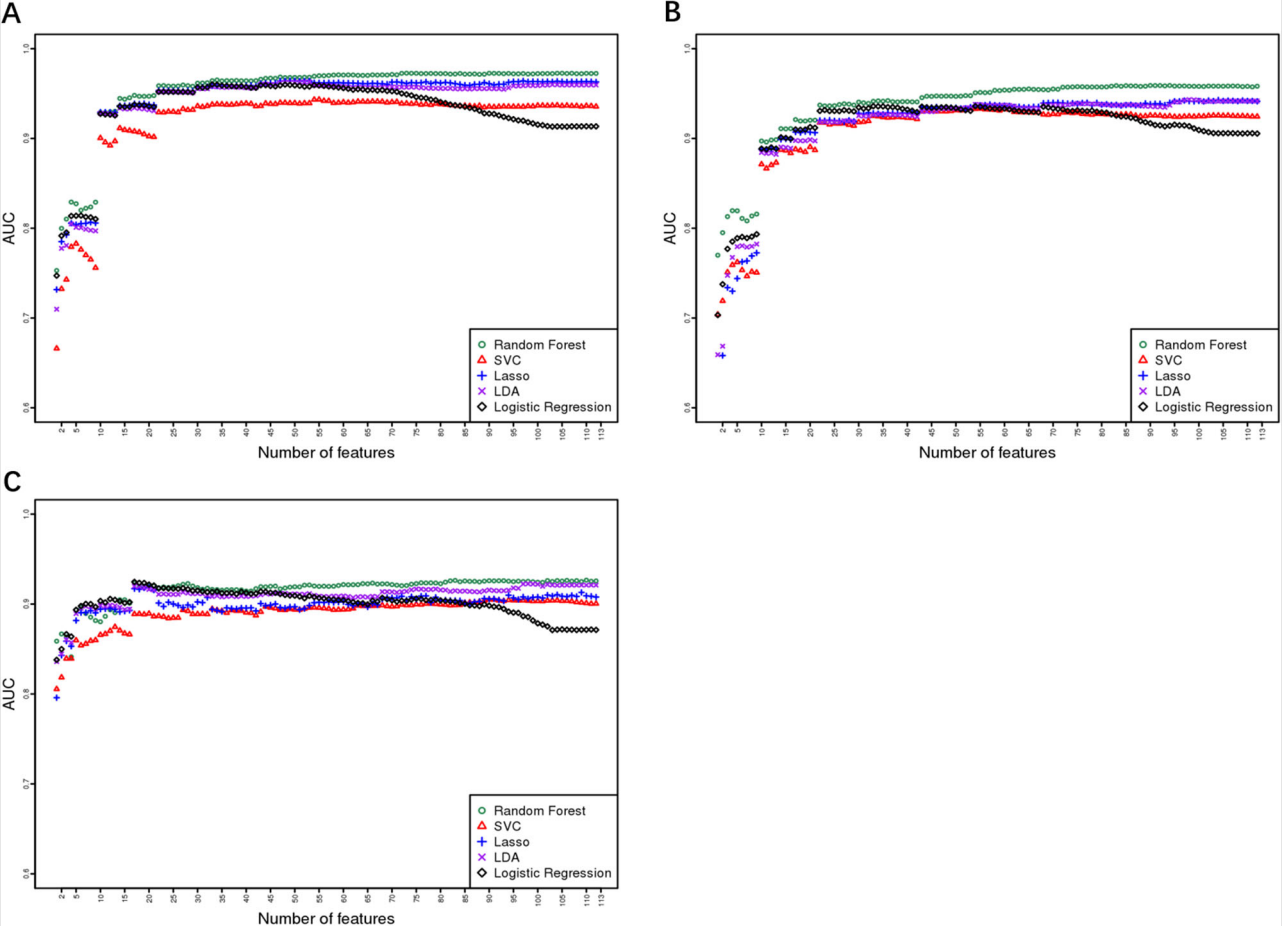
Forward feature selection results. **a** MDD diagnosis, **b** BPD diagnosis, and **c** HC diagnosis.

Forward feature selection results using the five aforementioned machine learning algorithms are presented in Fig. 1. The optimal number of features was determined at the point at which there was no further gain in AUC when more features were added. The random forest algorithm performed better than the other algorithms using the same number of features. LASSO and LDA behaved quite similar for the MDD and BPD models, but LDA outperformed LASSO for the HC model. Owing to its instinct mechanism, excessive features led to overfitting of the logistic regression model, and this model exhibited a gradual decline in performance as more features were added to the model. Therefore, the random forest algorithm was selected to generate the BDCC.

As shown in Table 2, the best performance of each machine learning algorithm was compared. The random forest algorithm performed the best but had the most features. Applying 74, 91 or 111 of the ADE questions is still time consuming, and thus, the number of questions was further reduced to make the BDCC feasible in clinical practice. Additional feature reduction was performed using the following criteria: (1) Unique questions were included to allow MDD, BPD, and HC diagnoses using the same model; (2) The AUCs for MDD, BPD, and HC were required to be >95%; (3) The number of questions was minimized.

**Table 2:** Precision of the machine learning algorithms and the BDCC.

	Random forest		SVR		LASSO		LDA		Logistic regression		BDCC	
	AUC	Features used	AUC	Features used	AUC	Features used	AUC	Features used	AUC	Features used	AUC	Features used
MDD	0.973	74/113	0.943	56/113	0.964	50/113	0.963	54/113	0.960	34/113	0.948	17/113
BPD	0.959	91/113	0.933	56/113	0.943	105/113	0.943	99/113	0.936	34/113	0.921	17/113
HC	0.927	111/113	0.905	91/113	0.918	21/113	0.923	99/113	0.925	18/113	0.923	17/113

*BDCC* Bipolar Diagnosis Checklist in Chinese, *SVR* support vector regression, *LASSO* least absolute shrinkage and selection operator, *LDA* linear discriminant analysis, *AUC* area under curve, *MDD* major depressive disorder, *BPD* bipolar disorder, *HCs* healthy controls

**Fig. 2:**
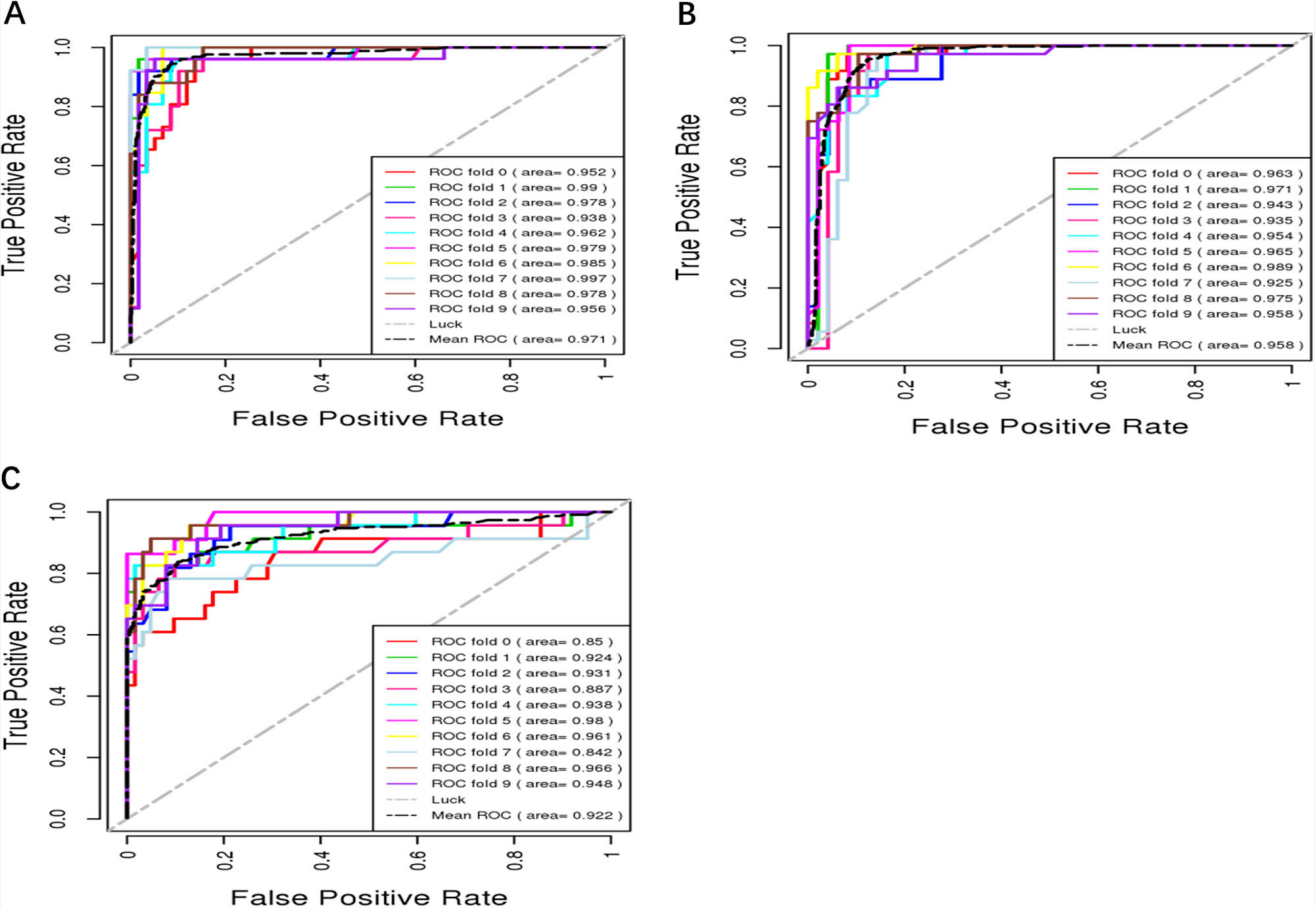
ROC curves of 10-fold cross-validation subsamples of the best random forest performance. **a** MDD diagnosis, **b** BPD diagnosis, and **c** HC diagnosis.

**Fig. 3:**
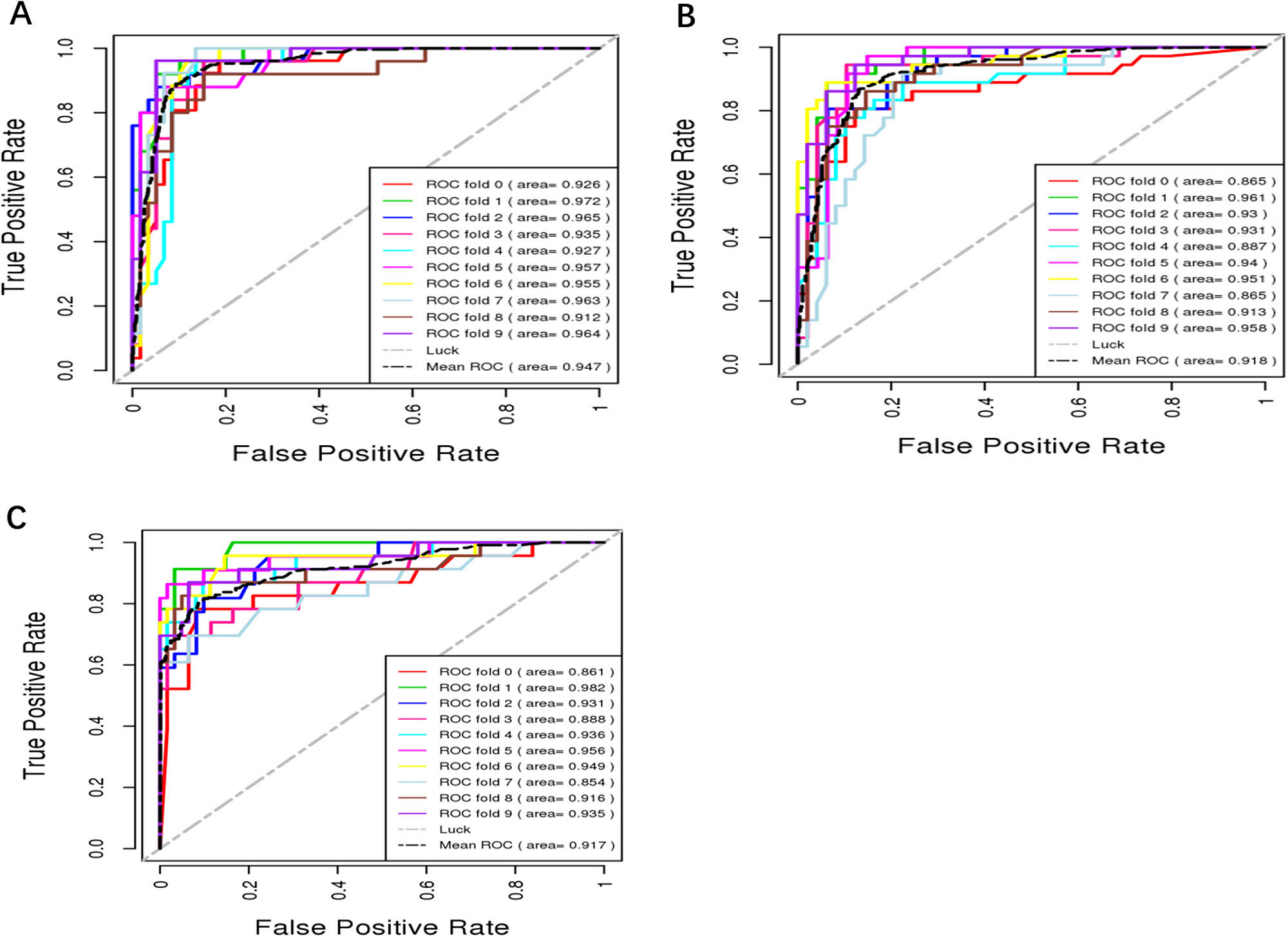
ROC curves of 10-fold cross-validation subsamples of the BDCC. **a** MDD diagnosis, **b** BPD diagnosis, and **c** HC diagnosis.

Ultimately, 17 questions were selected to comprise the BDCC. All these questions can be found in Table 1. The ROC curves of the 10-fold cross-validation subsamples of the best random forest performance and the BDCC performance are shown in Figs. 2 and 3. Both were robust, and the BDCC had 97.4% (0.948/0.973), 96.0% (0.921/0.933), and 99.6% (0.923/0.905) accuracy using only 23.0% (17/74), 18.7% (17/91), and 15.3% (17/111) of the items from the best random forest performance to diagnose MDD, BPD, and HC, respectively. Eventually, using only 15% (17/113) of the ADE items, BDCC had AUCs of 0.948, 0.921, and 0.923 for diagnosing MDD, BPD, and HC, respectively.

## Discussion

Utilizing five machine learning algorithms, we abbreviated the ADE^22^, a time-efficient record-keeping instrument typically used in research studies, using Chinese multicenter cohort data. The increased AUC reported herein compared with our previous work^6^ indicates the acceptability of this result and the validity of the Chinese version of the BDCC. In addition, the BDCC halves the time needed to collect clinical information. It takes more than 30 min to finish the ADE, whereas it takes only 10–15 min to complete the BDCC. Our current results reveal that the BDCC is as robust as the original version but more feasible to implement.

The 17 selected items of the BDCC fall into three categories: current clinical status (11 questions), lifetime clinical trials (5 questions including sudden onset and anger attacks of past depression, dysthymia, age at first use of antidepressant medication, and lifetime euphoria), and past psychiatry history (1 questionnaire on suicide attempts). These above categories correspond to diagnostic criteria, such as those of the DSM-IV.

In the domain of current clinical status, our results suggest that racing thoughts/flight of ideas (FOI), psychomotor agitation and irritability have highly significant correlations with BPD, which is generally in accordance with previous findings^23^. For lifetime traits, we referred to other studies describing more sudden onset^24^ and anger attacks^25^ among bipolar depressed patients than unipolar depressed patients and reporting that individuals with BPD feel more euphoria than those suffering from MDD^26^. Moreover, receiving antidepressant treatment at a relatively young age^27^ and frequently attempting suicide seem to be common among BPD patients. On the other hand, dysthymia corresponded to MDD rather than BPD^28^. Thus, the lifetime features of the BDCC may enhance its stability and feasibility for the diagnosis of BPD and MDD.

In addition, symptoms addressed by the BDCC may explicitly relate to the switch from MDD to BPD. For example, the severity of current mood elevation, addressed by the BDCC, is highly suggestive of BPD, and a previous study identified this symptom as a promising predictor of switching from MDD to BPD^29^. Higher severity of other manic symptoms, including flight of ideas (FOI), psychomotor agitation (PMA), anxiety, and irritability, was associated with a higher risk of switching^30^. Sudden onset of past depression is also a risk factor for switching^31^ from MDD to BPD. Thus, the BDCC may help in the early recognition of BPD.

### Limitations

This study has several limitations, including its cross-sectional nature and the available content of the data sets. Therefore, based on our existing work^10^, a prospective cohort study with a larger sample size will be conducted in the future. A classifier to distinguish bipolar II disorder (BD II) from bipolar I disorder (BD I) will be built by both retrospective and prospective analysis using a new cohort.

## Conclusion

In summary, the BDCC scale is a reasonable alternative diagnostic instrument for identifying BPD and MDD and it is a balance between time consuming and amount of questionnaire items optimized by machine learning. Our future study will focus on prospective validation of the BDCC scale.
